# Mucosal and salivary microbiota associated with recurrent aphthous stomatitis

**DOI:** 10.1186/s12866-016-0673-z

**Published:** 2016-04-01

**Authors:** Yun-ji Kim, Yun Sik Choi, Keum Jin Baek, Seok-Hwan Yoon, Hee Kyung Park, Youngnim Choi

**Affiliations:** Department of Oral Microbiology and Immunology, School of Dentistry and Dental Research Institute, Seoul National University, 101 Daehak-ro, Seoul, Jongno-gu 110-744 Republic of Korea; School of Biological Sciences, Seoul National University, Seoul, Republic of Korea; Department of Oral Medicine and Oral Diagnosis, School of Dentistry and Dental Research Institute, Seoul National University, 101 Daehak-ro, Seoul, Jongno-gu 110-744 Republic of Korea

**Keywords:** Recurrent aphthous stomatitis, Oral microbiota, Pyrosequencing

## Abstract

**Background:**

Recurrent aphthous stomatitis (RAS) is a common oral mucosal disorder of unclear etiopathogenesis. Although recent studies of the oral microbiota by high-throughput sequencing of 16S rRNA genes have suggested that imbalances in the oral microbiota may contribute to the etiopathogenesis of RAS, no specific bacterial species associated with RAS have been identified. The present study aimed to characterize the microbiota in the oral mucosa and saliva of RAS patients in comparison with control subjects at the species level.

**Results:**

The bacterial communities of the oral mucosa and saliva from RAS patients with active lesions (RAS, *n* = 18 for mucosa and *n* = 8 for saliva) and control subjects (*n* = 18 for mucosa and *n* = 7 for saliva) were analyzed by pyrosequencing of the 16S rRNA genes. There were no significant differences in the alpha diversity between the controls and the RAS, but the mucosal microbiota of the RAS patients showed increased inter-subject variability. A comparison of the relative abundance of each taxon revealed decreases in the members of healthy core microbiota but increases of rare species in the mucosal and salivary microbiota of RAS patients. Particularly, decreased *Streptococcus salivarius* and increased *Acinetobacter johnsonii* in the mucosa were associated with RAS risk. A dysbiosis index, which was developed using the relative abundance of *A. johnsonii* and *S. salivarius* and the regression coefficients, correctly predicted 83 % of the total cases for the absence or presence of RAS. Interestingly, *A. johnsonii* substantially inhibited the proliferation of gingival epithelial cells and showed greater cytotoxicity against the gingival epithelial cells than *S. salivarius.*

**Conclusion:**

RAS is associated with dysbiosis of the mucosal and salivary microbiota, and two species associated with RAS have been identified. This knowledge may provide a diagnostic tool and new targets for therapeutics for RAS.

**Electronic supplementary material:**

The online version of this article (doi:10.1186/s12866-016-0673-z) contains supplementary material, which is available to authorized users.

## Background

Recurrent aphthous stomatitis (RAS) is one of the most common oral mucosal disorders affecting at least 10 to 20 % of the general population [1]. RAS is characterized by the recurrent occurrence of well-circumscribed, single or multiple ulcers that are extremely painful and heal more slowly than traumatic ulcers of similar size [2]. Diverse factors, including genetic predisposition, immunologic disturbances, viral and bacterial infections, food allergies, vitamin and microelement deficiencies, systemic diseases, hormonal imbalance, mechanical injuries, and stress, have been suggested to trigger or to be associated with RAS [3]. However, the etiopathogenesis of RAS remains unclear. Consequently, no curative treatment is available and patient care primarily consists of symptomatic treatment [1].

Among the bacterial infections, the role of a *Streptococcus* strain (first identified as *S. sanguinis* but now reclassified as *S. oralis*) has been extensively studied since its isolation from a RAS lesion. Cross-reaction of anti-*Streptococcal* antibodies with autoantigens in the oral mucosa was proposed as an etiopathogenic mechanism of RAS, but no evidence to support this hypothesis has been found [4]. Later, lack of association between *S. oralis* and RAS was reported based on the less frequent detection of *S. oralis* in RAS than in healthy control samples [5]. Cross-reactive recognition of the mycobacterial and human heat shock protein 65-60 antigen by T cells of RAS patients has been reported, suggesting the role of *Mycobacterium tuberculosis* and autoreactive T cells in RAS [6]. Accumulated evidence supports the association between RAS and *Helicobacter pylori* infection, but the presence of *H. pylori* at the RAS lesions is controversial [7, 8]. In an attempt to discover the microorganisms present in RAS lesions, Marchini et al. [9] studied the mucosal microbiota in RAS patients using a culture-independent method. Due to limitations in the methods available at that time, only 57 and 38 phylotypes were defined from 10 RAS and 10 healthy subjects, respectively. Recently, the salivary microbiota in patients with inflammatory bowel disease, where RAS is one of the extraintestinal manifestations of inflammatory bowel disease [10], and the oral mucosal microbiota in RAS patients have been studied by high-throughput sequencing of the 16S rRNA genes [11, 12]. In addition, Terminal-Restriction Fragment Length Polymorphism analysis of bacterial 16S rRNA genes, the human oral microbe identification microarrays, and matrix-assisted laser desorption/ionization time-of-flight analysis have been applied to study the oral microbiota of RAS patients [13, 14]. Although all previous studies suggested that imbalances in the oral microbiota may be involved in the etiopathogenesis of RAS, no specific bacterial species associated with RAS have been identified. We previously characterized the murine oral microbiota to the species level by pyrosequencing [15]. Therefore, this study aimed to characterize the microbiota of the oral mucosa and saliva of RAS patients compared with control subjects at the species level. Pyrosequencing analysis successfully characterized the oral microbiota of RAS patients and identified two species associated with RAS risk.

## Results

### Subjects

The demographic data of RAS patients and control subjects included in the current study are summarized in Table 1. Nine males and nine females with the active lesions of minor RAS were included and the age distribution of the patients was 19 to 81 years. The mucosal sampling sites of RAS lesions included the tip of the tongue (*n* = 4), the buccal mucosa (*n* = 4), and the labial mucosa (*n* = 10). Twelve patients had a single ulcer and six patients had multiple ulcers on the tip of the tongue, labial mucosa, and soft palate. The control group included eight males and 10 females with an age distribution of 21 to 71 years. The mucosal sampling sites included the buccal mucosa (*n* = 11) and the labial mucosa (*n* = 7).

**Table 1:** The demographic data of the control subjects and RAS patients

	Control subjects (*n* = 18)	RAS patients (*n* = 18)
Gender	8 males, 10 females	9 males, 9 females
Age	43.6 ± 3.7	43.8 ± 3.9
Ulcer numbers	-	Single: 12 (66.7 %) Multiple: 6 (33.3 %)
Sampling sites	Lip labial mucosa: 7 (38.9 %), Buccal mucosa: 11 (61.1 %)	Lip labial mucosa: 10 (55.6 %), Buccal mucosa: 4 (22.2 %) Tongue tip: 4 (22.2 %),
Unstimulated salivary flow rates	0.48 ± 0.09 ml/minute	0.67 ± 0.08 ml/minute

### The alpha and beta diversities of the oral microbiota

**Fig. 1:**
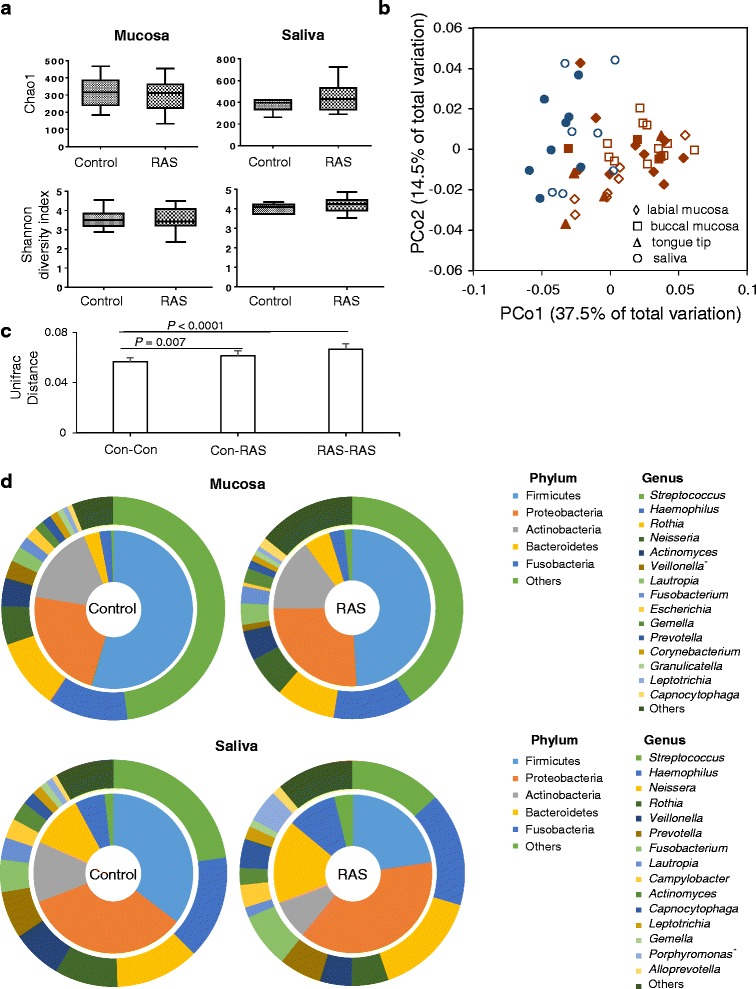
Comparison of mucosal and salivary microbiota between control and RAS. **a** The species richness estimated by Chao1 and Shannon diversity index are expressed using box and whisker plots. **b** PCoA plot generated using weighted Unifrac metric. The two components explained 52 % of variance. (unfilled symbols: control samples, filled symbols: RAS samples). **c** The intra- and intergroup Unifrac distances of mucosal communities were obtained using weighted metric. **d** Double pie charts present the mean relative abundance of dominant phyla (top 5) and genera (top 15). * denotes significant difference by Mann-Whitney U test (*P* < 0.01)

From the 51 communities, total 484,501 filtered reads (average 9500 reads per sample) with an average length of 479 bp were obtained, which resulted in greater than 99 % Good’s coverage for each sample. We first compared the alpha diversity between the control and RAS group. The species richness of the RAS microbiota estimated by Chao 1 was not significantly different from that of controls either in the mucosa (314 ± 19 vs. 292 ± 22) or in the saliva (377 ± 14 vs. 447 ± 33). The diversities of RAS microbiota determined by the Shannon index were also comparable to those of controls both in the mucosa (3.57 ± 0.11 vs. 3.56 ± 0.06) and in the saliva (4.02 ± 0.09 vs. 4.21 ± 0.1) (Fig. 1a).

UniFrac-based principal coordinate analysis (PCoA) to determine variation among the samples revealed that the microbiota profile was differentiated better by the anatomical sites, i.e., mucosal surfaces vs. saliva, than by disease. The different locations in the mucosa, i.e., tongue tip, labial, or buccal, did not show distinct clustering (Fig. 1b). Although PCoA clustering did not reveal clear separation between the control and RAS communities, the intergroup UniFrac distance (0.061 ± 0.001) was higher than the intragroup distance of controls (0.057 ± 0.001), suggesting a significant difference in the bacterial profile between control and RAS samples. In addition, a higher intragroup UniFrac distance in the RAS (0.067 ± 0.001) compared to the control group suggested the increased inter-subject variability for RAS lesions (Fig. 1c). In the salivary communities that reflect not only the diseased sites but also the healthy sites of patients, no significant differences were observed in intra- or intergroup UniFrac distances.

### Differences in oral microbiota composition between the control and RAS group

Next, the relative abundance of each taxon between the control and RAS group was compared. Although a total of 26 different phyla were identified from the mucosa samples, Firmicutes, Proteobacteria, Actinobacteria, Bacteroidetes, and Fusobacteria encompassed the majority of the sequences (>99 % in Controls and >97 % in RAS). The relative abundance of the major phyla observed in the control subjects was not significantly different from that of the RAS patients (Fig. 1d). However, the relative abundance of Streptophyta, a minor phylum, was significantly increased in the RAS group (*P* = 0.03). At the genus level, *Streptococcus* constituted almost half of the total mucosal microbiota in the control group and the other major genera included *Haemophilus*, *Rothia*, *Neisseria*, *Actinomyces*, and *Veillonella*. Compared to controls, the RAS mucosal communities contained significantly reduced abundance of *Veillonella* (Fig. 1d). Fourteen other genera also showed differences in the relative abundance (Table 2). At the species level, the abundance of several *Streptococcus*, including *S. salivarius*, *V. dispar*, *R. dentocariosa*, *A. odontolyticus*, and *Prevotella histicola* was decreased in the RAS mucosa. Instead, the abundance of *Acinetobacter oryzae*, *A. johnsonii*, *Capnocytophaga sputigena*, *N. oralis*, *Myxococcus xanthus*, *Ruminococcus gnavus*, and *Treponema denticola* was increased (Table 2).

**Table 2:** Relative abundance^a^ of taxa differently distributed between the controls and RAU in the mucosal microbiota

		Controls (*n* = 18)	RAU (*n* = 18)	*P* value
Genus	*Veillonella*	1.86 (0.49–10.44)	0.82 (0–3.33)	0.003
	*Acinetobacter*	0 (0–2.54)	1.04 (0–7.69)	0.001
	*DQ241813_g* (Flavobacteriaceae)^b^	0.05 (0–1.72)	0.14 (0–11.22)	0.04
	*Lachnoanaerobaculum*	0.08 (0.02–0.87)	0.04 (0–0.47)	0.017
	*Blautia*	0 (0–0.24)	0.02 (0–3.73)	0.01
	*Myxococcus*	0 (0–0.14)	0.04 (0–3.73)	0.031
	*Alloprevotella*	0.04 (0–0.22)	0.09 (0–0.49)	0.031
	*Pseudomonas*	0 (0–0.33)	0.02 (0–2.28)	0.027
	*Atopobium*	0.04 (0–0.44)	0 (0–0.42)	0.037
	*Ruminococcus_g6*	0 (0–0.55)	0.02 (0–0.86)	0.031
	*Faecalibacterium*	0 (0–0.09)	0.02 (0–0.58)	0.014
	*Staphylococcus*	0 (0–0.15)	0.02 (0–0.28)	0.009
	*Streptococcaceae_uc*	0.02 (0–0.06)	0 (0–0.04)	<0.0001
	*Flavobacterium*	0 (0–0)	0 (0–0.38)	0.047
	*Clostridium_g6*	0 (0–0)	0 (0–0.14)	0.047
Species	*Streptococcus salivarius*	4.84 (0.08–18.08)	0.61 (0–10.20)	0.001
	*Veillonella dispar*	1.63 (0.29–5.30)	0.60 (0–2.16)	0.003
	*Streptococcus parasanguinis*	1.12 (0–11.45)	0.06 (0–0.98)	0.001
	*Rothia dentocariosa*	0.53 (0.01–9.91)	0.10 (0–3.71)	0.034
	*Acinetobacter oryzae*	0 (0–2)	0.75 (0–5.88)	0.01
	*Actinomyces odontolyticus*	0.43 (0–1.73)	0.11 (0–1.45)	0.02
	*Capnocytophaga sputigena*	0.01 (0–0.53)	0.08 (0–3.26)	0.047
	*Acinetobacter johnsonii*	0 (0–0.54)	0.21 (0–1.86)	0.001
	*Streptococcus_uc*	0.14 (0.03–1.35)	0.05 (0–0.16)	0.001
	*FM997095_s* (*Streptococcus*)^b^	0.17 (0.01–0.91)	0.03 (0–1.25)	0.005
	*Neisseria oralis*	0 (0–0.16)	0.03 (0–1.83)	0.017
	*HQ757980_s* (*Streptococcus*)^b^	0.07 (0–1.15)	0 (0–2.32)	0.017
	*4P003152_s* (*Streptococcus*)^b^	0.06 (0–1.27)	0 (0–0.09)	0.006
	*Campylobacter concisus*	0.09 (0–0.52)	0.02 (0–0.14)	0.002
	*Prevotella histicola*	0.01 (0–2.29)	0 (0–0.29)	0.027
	*Myxococcus xanthus*	0 (0–0.14)	0.04 (0–3.73)	0.031
	*Streptococcus vestibularis*	0.02 (0–0.33)	0 (0–1.25)	0.001
	*Streptococcus lactarius*	0.02 (0–0.87)	0 (0–0.10)	0.031
	*4P002810_s* (*Prevotella*)^b^	0.05 (0–0.38)	0 (0–0.34)	0.024
	*Ruminococcus gnavus*	0 (0–0.53)	0.02 (0–0.86)	0.034
	*BABG01000051_s* (*Faecalibacterium*)^b^	0 (0–0.07)	0.01 (0–0.52)	0.016
	*Treponema denticola*	0 (0–0.10)	0.02 (0–0.14)	0.031
	*Streptococcaceae_uc_s*	0.02 (0–0.06)	0 (0–0.04)	<0.0001
	*FJ976422_s* (*Alloprevotella*)^b^	0 (0–0.17)	0.01 (0–0.14)	0.047

^a^Relative abundance expressed as the median and range

^b^The lowest taxonomic rank classified to which the unclassified genus or species belongs

The salivary microbiota of RAS tended to contain decreased Firmicutes and increased Proteobacteria among the major phyla, but the differences were not significant. At the phylum level, only SR1 showed a significant difference (0.01 ± 0.004 vs 0.6 ± 0.5, *P =* 0.02). Among the top 15 genera shown in Fig. 1d, the RAS salivary microbiota was populated by a significantly increased *Porphyromonas*, and the relative abundance of three other genera was also increased (Table 3). At the species level, the abundance of *S. salivarius* was decreased in the RAS samples. Instead, the abundance of *N. flava*, *N.* sicca, *C. gingivalis*, *C. sputigena*, *Aggregatibacter segnis*, *Abiotrophia defectiva*, and unclassifieded *Porphyromonas* species (*FM995684_s* and *Porphyromonas_uc*) was increased (Table 3).

**Table 3:** Relative abundance^a^ of taxa differently distributed between the controls and RAU in the salivary microbiota

		Controls (*n* = 7)	RAU (*n* = 8)	*P* value
Genus	*Porphyromonas*	0.40 (0.17–2.65)	4.51 (0.88–12.27)	0.006
	*GU410548_g* (SR1)^b^	0.01 (0–0.02)	0.06 (0–4.72)	0.021
	*Abiotrophia*	0 (0–0)	0.04 (0–0.28)	0.014
	*Streptococcaceae_uc*	0 (0–0.02)	0.03 (0–0.04)	0.029
Species	*Streptococcus salivarius*	2.18 (0.76–10.42)	0.74 (0.03–3.84)	0.021
	*Neisseria flava*	0.06 (0–1.16)	0.53 (0–6.99)	0.04
	*Capnocytophaga gingivalis*	0.21 (0.06–0.62)	0.68 (0.04–3.50)	0.029
	*Aggregatibacter segnis*	0.02 (0–0.19)	0.42 (0–2.65)	0.021
	*Capnocytophaga sputigena*	0.06 (0–0.20)	0.38 (0.06–1.62)	0.004
	*FM995684_s* (*Porphyromonas*)^b^	0 (0–0.27)	0.30 (0–3.91)	0.029
	*4P003196_s* (*Actinomyces*)^b^	0.03 (0.01–0.42)	0.14 (0.10–2.09)	0.04
	*Neisseria sicca*	0 (0–0.25)	0.17 (0.02–1.37)	0.006
	*Porphyromonas_uc*	0 (0–0.03)	0.06 (0–0.55)	0.04
	*Abiotrophia defectiva*	0 (0–0)	0.03 (0–0.28)	0.04
	*4P004176_s* (SR1)^b^	0 (0–0.01)	0.03 (0–0.23)	0.021
	*Streptococcaceae_uc_s*	0 (0–0.02)	0.03 (0–0.04)	0.029

^a^Relative abundance expressed as the median and range

^b^The lowest taxonomic rank classified to which the unclassified genus or species belongs

### Identification of bacterial species associated with RAS

**Fig. 2:**
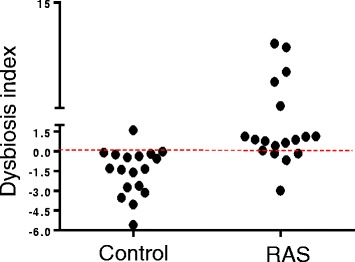
Dysbiosis index of RAS. A dysbiosis index was defined as 5.35 × [*A. johnsonii*] - 0.309 × [*S. salivarius*] using the relative abundance of *A. johnsonii* and *S. salivarius* in the mucosa where 5.35 and -0.309 are the regression coefficients. The dysbiosis index of 18 control samples and 18 RAS samples are shown. The dotted line indicates cutoff for RAS

We explored if such changes in the abundance of bacterial species is associated with RAS risk. A logistic regression analysis of the top 100 species in the mucosal microbiota using a forward method revealed that the abundance of *S. salivarius* was associated with a reduced RAS risk (OR 0.734 per 1 % increase, CI 95 % 0.565-0.954, *P =* 0.02), and the abundance of *A. johnsonii* was associated with an increased RAS risk (OR 211 per 1 % increase, CI 95 % 1618-2.7E4, *P =* 0.03). None of the species in the salivary microbiota showed significant association with RAS. A dysbiosis index was defined as 5.35 × [*A. johnsonii*] - 0.309 × [*S. salivarius*] using the relative abundance of *A. johnsonii* and *S. salivarius* in the mucosa where 5.35 and -0.309 are the regression coefficients. The dysbiosis index was significantly associated with RAS risk (OR 2.76. CI 95 % 1.26-6.05, *P* = 0.01) and correctly predicted 83 % of the total cases for the absence or presence of RAS (94 % of control and 72 % of RAS, Fig. 2).

**Fig. 3:**
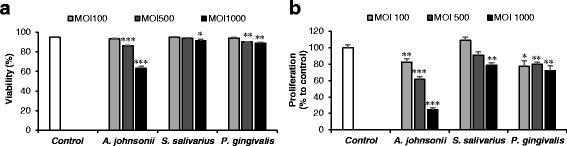
The effect of RAS-associated bacterial species on the viability and proliferation of human oral epithelial cells. HOK-16B cells were infected with *A. johnsonii*, *S. salivarius*, and *P. gingivalis* at MOIs of 100, 500, and 1000 for 24. The viability (**a**) and the number of live HOK-16B cells (**b**) in six wells from two independent experiments were determined by trypan blue exclusion and compared with control cells without bacterial infection. *: *P* < 0.05, **: *P* < 0.01, ***: *P* < 0.001

To understand the potential role of bacteria in the etiopathogenesis of RAS, the effects of two RAS-associated species on the viability and proliferation of oral epithelial cells were examined. *P. gingivalis*, a periodontal pathogen that has been reported to inhibit wound healing in an *in vitro* scratch assay [16], was used as a control. *A. johnsonii* showed greater cytotoxicity against HOK-16B cells than *S. salivarius* that showed low levels of cytotoxicity only at MOI 1000 (Fig. 3a). Interestingly, *A. johnsonii* substantially inhibited the proliferation of HOK-16B cells in a dose dependant manner (Fig. 3b).

## Discussion

In this study, we showed that imbalances in the mucosal and salivary microbiota are associated with RAS. Among the top 15 genera observed in the mucosa of control subjects, 13 except for *Escherichia* and *Lautropia* were common to the major genera characterized in normal adults enrolled in the HMP. Similarly, 14 genera except for *Lautropia* out of the top 15 genera observed in the saliva were common to the HMP [17], defining them as the healthy ‘core microbiota’ of oral microbial communities. Although most of the genus members of the normal flora were shared between Koreans and the HMP subjects, the order of the genus composition and the relative abundance of the major phyla were different (Additional file 1: Figure S1).

Among the 15 species/phylotypes significantly decreased in the RAS mucosa compared to the controls, nine species including *S. salivarius*, *S. parasanguinis*, *S. peroris*, *S. vestibularis*, *S. lactarius*, *V. dispar*, *Rothia dentocariosa*, *Campylobacter concisus*, *Actinomyces odontolyticus*, and *P. histicola* belonged to those defined as the normal flora of the oral mucosa [1, 17], and the six unclassified phylotypes also belonged to *Streptococcus* and *Prevotella*. In contrast, the seven species that were significantly increased in the RAS mucosa did not belong to the normal oral mucosal flora. *A. oryzae* and *N. oralis* have recently been isolated from wild rice and healthy gingival plaque, respectively [18, 19]. *Myxococcus xanthus* is a ubiquitous soil bacterium [20]. *A. johnsonii* is known to be a member of the skin flora [21]. *A. johnsonii*, the species associated with an increased RAS risk, has been isolated from clinical samples in association with bacteremia [22]. *R. gnavus* is a member of the gut flora [23]. Interestingly, an increase in *R. gnavus* in the gut flora is associated with Crohn’s disease [24]. In the salivary microbiota of RAS patients, the decrease in *S. salivarius* and the increase in *Capnocytophaga sputigena* were common to the changes observed in the mucosal microbiota. *Capnocytophaga* is normally found in the oral cavity but considered as an opportunistic pathogen involved in various infections, including endodontic infections, emphysema, and bacteremia [25–27]. Collectively, these results indicate decreases in the members of healthy core microbiota but increases in rare species in the mucosal and salivary microbiota of RAS patients. Particularly, a decrease in *S. salivarius* and an increase in *A. johnsonii* in the mucosa were associated with increased RAS risk.

Hijazi et al. reported changes in the relative abundance of only five unclassified genera in ulcerated sites of RAS patients compared with control subjects [12], probably due to the smaller size of the subjects and the smaller number of reads per sample compared to the current study. Because all of the reported genera are unclassified, it is difficult to appreciate the biological relevance of the changes in those. However, the increase of Porphyromonadaceae and decrease of Streptococcaceae in RAS reported by Hijazi et al. may agree with the increase of *Porphyromonas* in the saliva and decrease of many streptococcal species in the mucosa observed in the current study. The decrease in *Streptococcus* was also common in two other studies [5, 10]. The decrease of *Veillonella* in the RAS oral mucosa coincides with the study by Seoudi et al. [14]. The abundance of *S. oralis* was not different between the control and RAS groups, confirming the lack of association of *S. oralis* with RAS [5]. Either *H. pylori* or *M. tuberculosis*, the species proposed as bacterial etiology of RAS [6, 7], was not detected in any subjects of the current study.

Some diseases are associated with changes in microbial diversity. For example, periodontitis is associated with the increased diversity of plaque bacteria, while Crohn’s disease is associated with the reduced diversity of colonic microbiota [28, 29]. RAS was not associated with changes in the alpha-diversity of the mucosal or salivary microbiota, which agrees with previous studies [11, 13]. However, increased inter-subject variability of the mucosal microbiota was observed in the RAS patients. The increased inter-subject variability was not attributed to the different sampling sites, because the intragroup distance of RAS was still significantly higher than that of control group after removing the tongue tip samples (0.066 ± 0.002 vs. 0.057 ± 0.001, *P* = 0.0002). A similar high inter-sample variability has been reported in chemotherapy-related oral mucositis lesions [30]. The increased inter-subject variability may underlie the increased number of phylotypes characterized in the RAS mucosa in a study by Marchini et al. in which the samples from 10 RAS patients were pooled [9].

The dysbiosis index developed using the relative abundance of *A. johnsonii* and *S. salivarius* correctly predicted 83 % of the total cases for the absence or presence of RAS. This is a cross-sectional study, and there is no evidence that the bacterial species increased at the RAS lesions have a role in the initiation or progression of the disease. However, the lessons from the study of dental caries and periodontitis suggest that the bacterial species increased at the lesions may include the major pathogenic bacteria of the disease. Interestingly, *A. johnsonii* substantially inhibited the proliferation of gingival epithelial cells and showed increased cytotoxicity against epithelial cells. It has been reported that the relative abundance of *Streptococcus* was negatively associated with the concentrations of IL-1β and IL-8 in saliva [11]. Therefore, the imbalance of *A. johnsonii* and *S. salivarius* could contribute to ulceration, delayed healing, and severe pain caused by inflammatory cytokines, all of which are associated with RAS. Further host cell-microbe interactions are currently being studied.

When the imbalance between the healthy species such as *S. salivarius* and the potentially harmful species such as *A. johnsonii* is confirmed as the etiology of RAS, either probiotic application of *S. salivarius* or antibiotics that selectively kill harmful species but not healthy species may provide a cure for RAS by restoring the balance.

The current study has several limitations. First, the mucosal specimens of the control subjects were sampled from the labial and buccal mucosa, while the sampling sites from the RAS patients also included the tip of the tongue. Second, the limited number of total cases requires further study in larger cohorts and also in diverse populations, considering the differences in the relative abundance of major phyla comprising healthy microbiota between Koreans and the HMP subjects. Third, the non-ulcer sites of RAS patients were not studied. According to the study by Bankvall et al., the mucosal microbiota at the non-ulcer sites of RAS patients was different from that of controls, and the differences were most profound in patients who had lesions during sampling [13]. We originally designed the study to compare the mucosal microbiota in the lesions of RAS patients also with that in the post-healing sites. However, only four patients re-visited the clinic for additional sampling after healing, and those samples were not included in the current study. Longitudinal studies in the future will provide valuable evidence for the role of bacteria in the ediopathogenesis of RAS.

## Conclusion

Pyrosequencing analysis successfully characterized the oral microbiota of RAS patients compared with healthy controls at the species level. The mucosal microbiota of RAS lesions are characterized as decreases in the members of healthy core microbiota but increases of rare species, and a decrease in *S. salivarius* and an increase in *A. johnsonii* are associated with RAS risk. These findings may provide a diagnostic tool and new targets for the therapeutic management of RAS.

## Methods

### Ethics, consent and permissions

This study was performed according to the Declaration of Helsinki and conformed to the STROBE guidelines. The protocol was approved by the institutional review board at the Seoul National University Dental Hospital (CRI 12018). Written informed consent was obtained from all subjects.

### Study population and sample collection

Twenty patients with minor RAS active lesions who visited the Oral Medicine Clinic at the Seoul National University Dental Hospital from February 2013 to January 2014 and 20 control subjects without oral mucosal disorders were enrolled. Subjects who had received antibiotics or steroid within the last month, and patients with xerostomia (unstimulated whole salivary flow rate <0.1 ml/min) were excluded. Subjects with other oral mucosal diseases (Candida count > 1000 colony forming unit/ml, hematologic deficiency related diseases) or systemic diseases that involve oral ulcers were also excluded. We also compared the microbiota in the saliva of RAS, which reflects the microbiota in both the healthy and diseased sites of patients, with that of control subjects. All subjects were asked to avoid eating and antiseptic mouthwashes for two hours before sampling. For the mucosa sampling, a sterilized 20 mm × 20 mm polyvinylidene difluorid membrane (Korea, Seoul, Korea) was placed on the largest ulcerated area of patients for 30 s. Samples were taken from the labial or buccal mucosa of healthy subjects. A minimum of 2 ml unstimulated whole saliva samples were collected by a spit method.

### DNA extraction, 16S rRNA gene amplification, and pyrosequencing

**Fig. 4:**
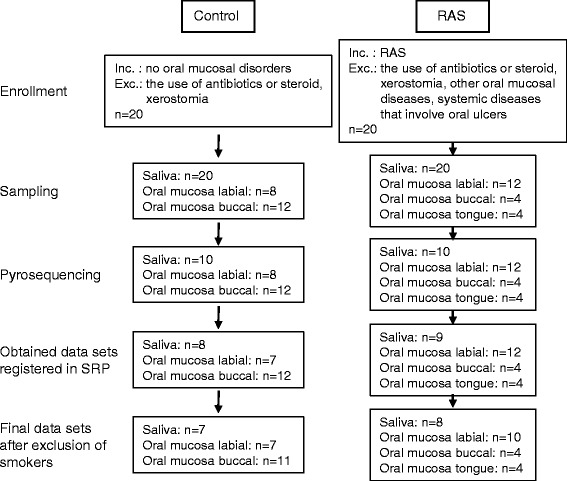
A flow chart from the enrollment of subjects to the acquisition of final data sets

Genomic DNA was isolated from the membranes and the pellets of centrifugated saliva using the PowerSoil DNA Isolation Kit (MO BIO Laboratories, Carlsbad, CA, USA). Forty mucosal (*n* = 20 and *n* = 20 for Controls and RAS, respectively) and 20 salivary (*n* = 10 and *n* = 10 for Controls and RAS, respectively) samples were subjected to pyrosequencing analysis. The gDNA was amplified using primers targeting the V1 to V3 hypervariable regions of bacterial 16S rRNA gene, and the PCR products were sequenced according to the previously described method [15] using a 454 GS FLX Titanum Sequencing System (Roche, Branford, CT, USA). Both the 16S rRNA gene amplification and sequencing were performed at ChunLab Inc. (Seoul, Korea). Out of the 60 samples analyzed, we successfully obtained data sets for 39 mucosal (*n* = 19 and *n* = 20 for Controls and RAS, respectively) and 17 salivary (*n* = 8 and *n* = 9 for Controls and RAS, respectively) microbiota communities. Four samples failed in pyrosequencing due to insufficient amplification of the 16S rRNA genes, although all DNA samples passed the quality control. Because communities from smokers are excluded from the final data set, the current study includes only 36 mucosal (*n* = 18 and *n* = 18 for Controls and RAS, respectively) and 15 salivary (*n* = 7 and *n* = 8 for Controls and RAS, respectively) microbiota communities. The pyrosequencing data, including those from smokers, are available in the SRP database under the accession number SRP049562. The results of data analysis including smokers are also provided as Additional file 1 (Figs. 2 and 3 and Tables 1, 2 and 3). The entire process from the enrollment of subjects to the acquisition of final data sets is illustrated as a flow chart (Fig. 4).

### Pyrosequencing data analysis

The basic analysis was conducted according to previously published descriptions [15]. After removing PCR primer sequences, any reads containing two or more ambiguous nucleotides or reads shorter than 300 bp were discarded. Chimera sequences detected by the Bellerophone method [31] were also removed. The taxonomic classification of each read was assigned against the EzTaxon database-e (http://www.ezbiocloud.net/eztaxon) [32]. The species richness and diversity index were calculated using the Ribosomal RNA database project’s pyrosequencing pipeline (http://pyro.cme.msu.edu). The cutoff value for assigning a sequence to the same phylotype was ≥ 97 % similarity. Random subsampling was conducted to equalize variation in the read counts among the samples. The overall phylogenetic distance between communities was estimated using the weighted Fast UniFrac [33] and was visualized using PCoA. In addition, the pyrosequencing data of buccal mucosa and saliva published by The Human Microbiome Project (HMP) Consortium [34] were also analyzed to compare with the control subjects of the current study.

### Bacterial and epithelial cell culture

Because isolation of bacteria from the patients was not included in the original protocol approved by the institutional review board, type strain was used for additional functional study. *A. johnsonii* KCTC 12405 (Korean Collection for Type Culture, Daejeon, Korea) was cultured in BHI medium at 30 °C and aerobic atmosphere. *S. salivarius* KCTC 5512 (KCTC) and *P. gingivalis* ATCC 33277 (American Type Culture Collection, Manassas, VA, USA) were cultured in ATCC medium 188 and BHI medium supplemented with 5 μg/ml hemin and 10 μg/ml vitamin K, respectively, at 37 °C under an anaerobic atmosphere. Bacteria in log phase growth were harvested and bacterial concentrations were determined by flow cytometry [35]. Immortalized human oral keratinocyte HOK-16B cells originated from the retromolar gingival tissue [36] were maintained in keratinocyte growth-culture medium (Clonetics, San Diego, CA, USA) containing supplementary growth factors. HOK-16B cells plated into 48-well plates at 4x10^4^ cells/well in triplicate were cultured for 24 h and then infected with bacteria at the multiplicity of infection (MOI) 0, 100, 500, and 1000 as previously described [37]. After 24 h of co-culture at 37 °C in a water-saturated atmosphere of 95 % air and 5 % CO_2_, cells were harvested, including the dead cells in the supernatant. The viability and total number of live cells in each well were determined by trypan blue exclusion under a microscope.

### Statistics

The data are presented as the mean ± the standard errors of means, unless described otherwise. The differences in relative abundance and in UniFrac distances between the two groups were determined with the Mann-Whitney U-test and t-test, respectively. The association of bacterial species with RAS risk was determined with a logistic regression analysis. Differences in the viability and proliferation between control and infected cells were analyzed by t-test. All statistics were performed using the SPSS Statistics19 software (SPSS Inc., Chicago, IL, USA). Significance was set at *P* < 0.05.

## Additional file

Additional file 1: The comparison of the oral mucosa and saliva microbiota between the control subjects of current study and HMP subjects at the phylum level. (PDF 428 kb)

## Abbreviations

HMP: Human Microbiome Project
PCoA: principal coordinate analysis
RAS: recurrent aphthous stomatitis
