# Supplementary material for: Mucosal and salivary microbiota associated with recurrent aphthous stomatitis
Source: BMC Microbiol. 2016 Apr 1;16:57. doi: 10.1186/s12866-016-0673-z (PMC4818471; doi:10.1186/s12866-016-0673-z)
Supplement: Additional file 1: — The comparison of the oral mucosa and saliva microbiota between the control subjects of current study and HMP subjects at the phylum level. (PDF 428 kb) [file 12866_2016_673_MOESM1_ESM.pdf]

Figure S1

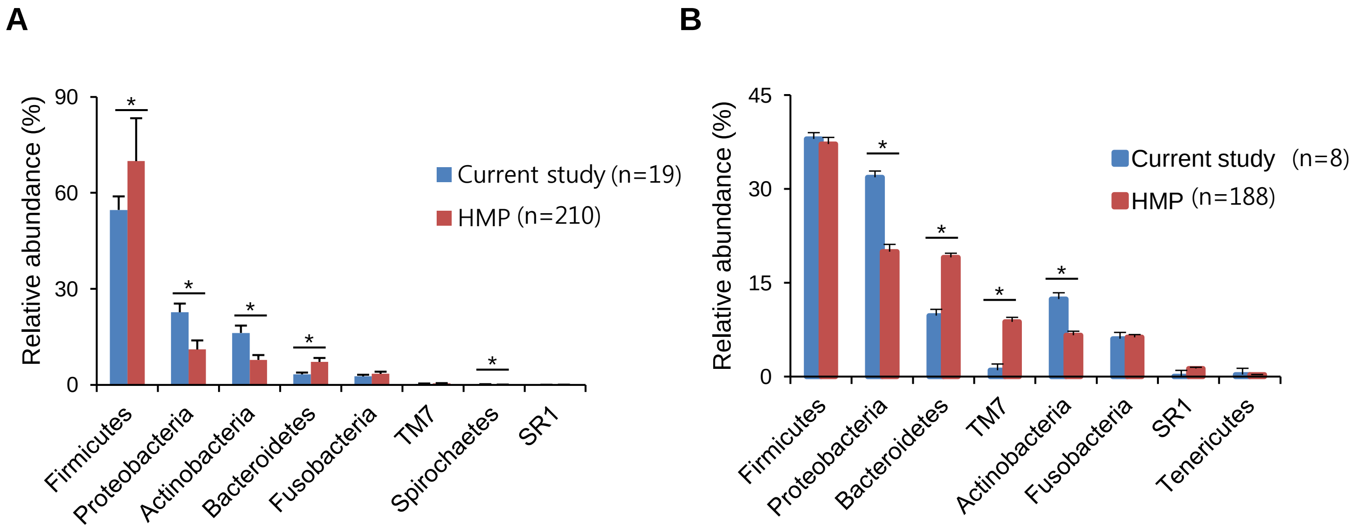

Dominant phyla observed in the mucosa (A) and saliva (B) of control subjects were compared with those published by Human Microbiome Project Consortium.

Figure S2

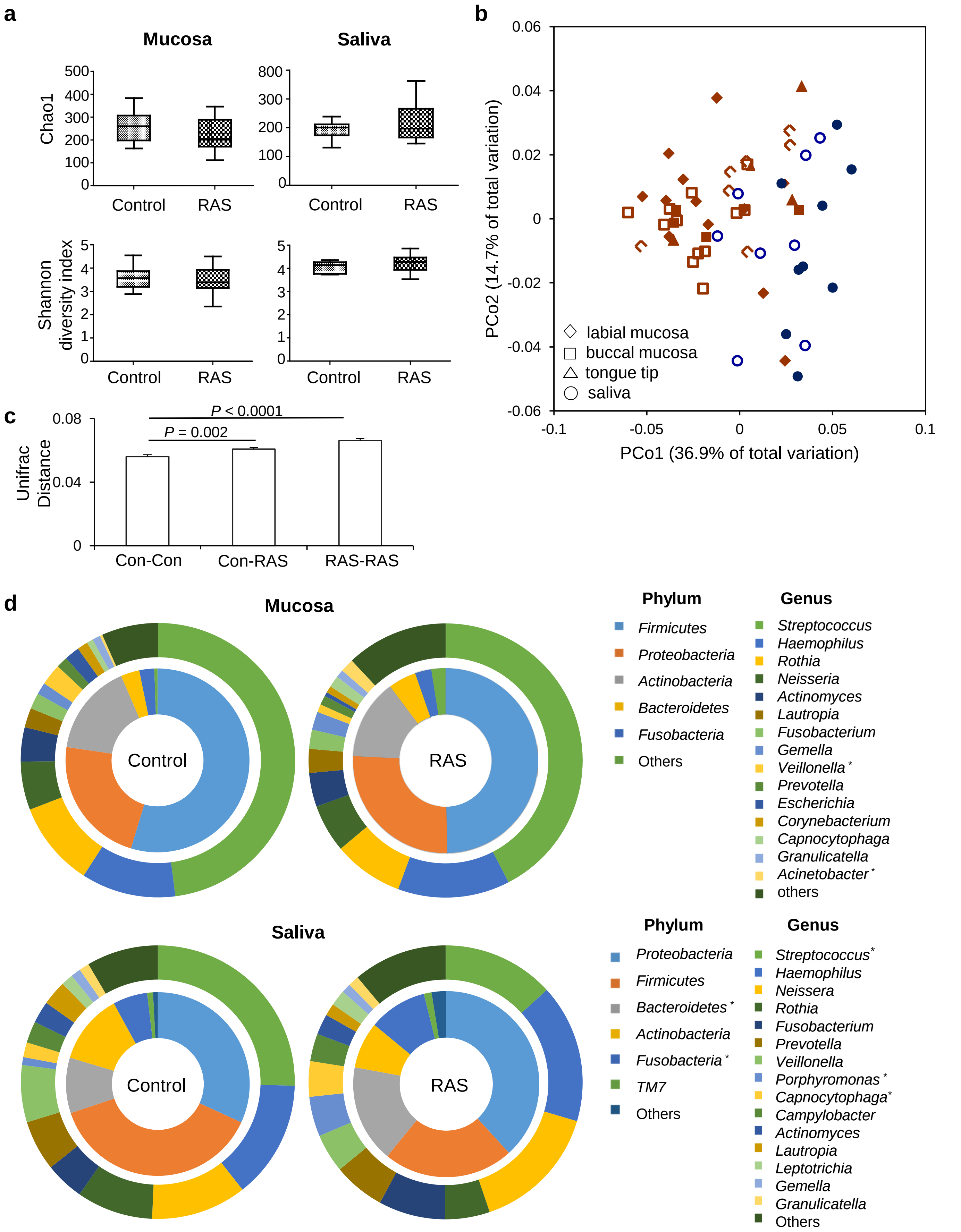
a
Mucosa
Saliva
Chao1
500
400
300
200
100
0
800
300
200
100
0
Control
RAS
Shannon diversity index
5
4
3
2
1
0
Control
RAS
b
0.06
0.04
0.02
0
-0.02
-0.04
-0.06
-0.1
-0.05
0
0.05
0.1
PCo2 (14.7% of total variation)
PCo1 (36.9% of total variation)
labial mucosa
buccal mucosa
tongue tip
saliva
c
Unifrac Distance
0.08
0.04
0
P < 0.0001
P = 0.002
Con-Con
Con-RAS
RAS-RAS
d
Mucosa
Control
RAS
Phylum
Firmicutes
Proteobacteria
Actinobacteria
Bacteroidetes
Fusobacteria
Others
Genus
Streptococcus
Haemophilus
Rothia
Neisseria
Actinomyces
Lautropia
Fusobacterium
Gemella
Veillonella *
Prevotella
Escherichia
Corynebacterium
Capnocytophaga
Granulicatella
Acinetobacter *
others
Saliva
Control
RAS
Phylum
Proteobacteria
Firmicutes
Bacteroidetes *
Actinobacteria
Fusobacteria *
TM7
Others
Genus
Streptococcus *
Haemophilus
Neissera
Rothia
Fusobacterium
Prevotella
Veillonella
Porphyromonas *
Capnocytophaga *
Campylobacter
Actinomyces
Lautropia
Leptotrichia
Gemella
Granulicatella
Others

Figure S3

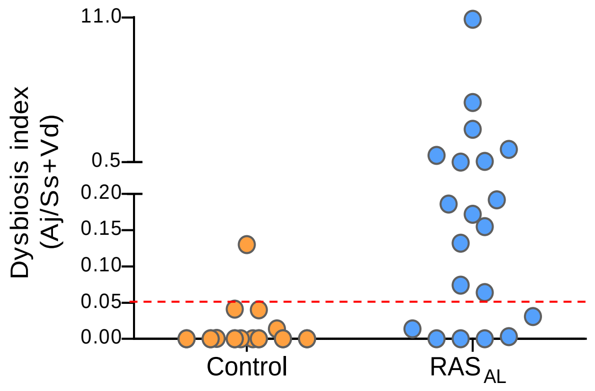

Dysbiosis index of RAS A logistic regression analysis of the mucosal microbiota using a forward method revealed that the abundance (per % increase) of *S. salivarius* was associated with a reduced RAS risk (odds ratio [OR] 0.691, 95% confidence interval [95% CI] 0.526-0.909, *P*=0.008). The analysis using a backward method revealed two species. The abundance of *V. dispar* was associated with a reduced RAS risk (OR 0.124, CI 95% 0.024-0.646, *P*=0.013), and the abundance of *A. johnsonii* was associated with an increased RAS risk (OR 7547, CI 95% 4.9-1.2E7, *P*=0.017). A dysbiosis index was defined as the relative abundance of *A. johnsonii* divided by the relative abundance of *S. salivarius* plus *V. dispar* in the mucosa.

Table. 1 . The demographic data of control subjects and RAS patients.

	Control subjects (n = 19)	RAS patients (n = 20)
Sex	9 males, 10 females	11 males, 9 females
Age	44.1±3.5	44.4±3.5
Smoking history	1 (5.3%)	3 (15%)
Ulcer numbers	-	Single: 13 (65%)<br>Multiple: 7 (35%)
Sampling sites	Lip labial mucosa: 7 (36.8%),<br>Buccal mucosa: 12 (63.2%)	Lip labial mucosa: 4 (20%),<br>Buccal mucosa: 13 (65%)<br>Tongue tip: 3 (15%),
Unstimulated salivary flow rates	0.46±0.08 ml/minute	0.63±0.07 ml/minute

Table 2. Relative abundance of taxa differently distributed between controls and RAU in the mucosal microbiota.

		Controls (n= 19)	RAU (n= 20)	*P* value
Genus	*Veillonella*↓	1.82 (0.49-10.44)	0.82 (0-3.33)	0.002
	Acinetobacter↑	0 (0-2.54)	0.79 (0-7.69)	0.001
	Campylobacter↓	0.22 (0-1.07)	0.08 (0-2.11)	0.035
	Lachnoanaerobaculum↓	0.09 (0.02-0.87)	0.04 (0-0.47)	0.007
	Blautia↑	0 (0-0.24)	0.02 (0-3.73)	0.002
	Myxococcus↑	0 (0-0.14)	0.04 (0-3.73)	0.006
	Pseudomonas↑	0 (0-0.33)	0.02 (0-2.28)	0.011
	Atopobium↓	0.04 (0-0.44)	0 (0-0.42)	0.025
	Ruminococcus_g6↑	0 (0-0.55)	0.02 (0-0.86)	0.012
	Moryella↓	0.04 (0-0.45)	0 (0-0.17)	0.001
	Faecalibacterium↑	0 (0-0.09)	0.01 (0-0.58)	0.012
	Staphylococcus↑	0 (0-0.15)	0.02 (0-0.28)	0.004
	Streptococcaceae_uc↓	0.02 (0-0.06)	0 (0-0.04)	< 0.0005
	Flavobacterium↑	0 (0-0)	0 (0-0.38)	0.002
Species	*Streptococcus salivarius*↓	4.51 (0.08-18.08)	0.52 (0-10.2)	< 0.0005
	Veillonella dispar↓	1.54 (0.29-5.30)	0.49 (0-2.16)	0.001
	Streptococcus parasanguinis↓	1.32 (0-11.45)	0.06 (0-0.98)	0.001
	Acinetobacter oryzae↑	0 (0-1.1)	0.58 (0-5.88)	0.006
	Actinomyces odontolyticus↓	0.41 (0-1.73)	0.11 (0-1.45)	0.009
	Veillonella atypica↓	0.06 (0-4.69)	0 (0-0.97)	0.032
	Prevotella melaninogenica↓	0.12 (0.05-0.85)	0.07 (0-0.69)	0.038
	Acinetobacter johnsonii↑	0 (0-0.54)	0.2 (0-1.86)	0.001
	FM997095_s↓	0.2 (0.01-1.35)	0.03 (0-1.25)	0.003
	Streptococcus_uc↓	0.15 (0.03-1.35)	0.05 (0-0.16)	< 0.0005
	Neisseria oralis↑	0 (0-0.81)	0.02 (0-1.83)	0.025
	HQ757980_s↓	0.09 (0-1.15)	0 (0-2.32)	0.008
	Capnocytophaga granulosa↓	0.03 (0-2.55)	0 (0-0.79)	0.018
	4P002811_s↓	0.06 (0-1.4)	0.01 (0-0.42)	0.034
	4P003152_s↓	0.06 (0-1.27)	0 (0-0.09)	0.001
	Streptococcus peroris↓	0.075 (0-0.56)	0.003 (0-0.4)	0.027
	Campylobacter concisus↓	0.1 (0-0.51)	0.02 (0-0.14)	0.001
	4P003193_s↓	0.02 (0-2.07)	0 (0-0.47)	0.027
	Prevotella histicola↓	0.01 (0-2.29)	0 (0-0.29)	0.007
	Lachnoanaerobaculum orale↓	0.07 (0.01-0.53)	0.02 (0-0.45)	0.028
	Streptococcus vestibularis↓	0.02 (0-0.33)	0 (0-1.25)	< 0.0005
	Streptococcus lactarius↓	0.03 (0-0.87)	0 (0-0.1)	0.011
	Blautia wexlerae↑	0 (0-0.24)	0.02 (0-3.21)	0.001
	Prevotella salivae↓	0.01 (0-0.49)	0 (0-0.96)	0.007
	DQ447842_s↓	0.05 (0-0.32)	0.006 (0-0.11)	0.035
	4P002810_s↓	0.05 (0-0.38)	0 (0-0.34)	0.013
	Ruminococcus gnavus↑	0 (0-0.53)	0.02 (0-0.86)	0.014
	Stomatobaculum longum↓	0.03 (0-0.45)	0 (0-0.17)	0.001
	Bacteroides fragilis↑	0 (0-0.29)	0.004 (0-0.43)	0.013
	BABG01000051_s↑	0 (0-0.07)	0.004 (0-0.52)	0.007
	Streptococcaceae_uc_s↓	0.02 (0-0.06)	0 (0-0.04)	0< 0.0005

Table 3. Relative abundance of taxa differently distributed between controls and RAU_{AL} in the salivary microbiota.

		Controls (n= 8)	RAU_{AL} (n= 9)	*P* value
Genus	*Streptococcus* ↓	25.78 (12.10-44.24)	13.05 (3.5-26.86)	0.034
	Porphyromonas ↑	0.51 (0.17-2.65)	3.43 (0.23-12.27)	0.016
	Capnocytophaga ↑	0.93 (0.19-6.43)	2.56 (1.55-10.43)	0.043
	GU410548_g ↑	0.02 (0-0.03)	0.07 (0-4.72)	0.014
	Abiotrophia ↑	0 (0-0.09)	0.04 (0-0.28)	0.017
	Escherichia ↓	0.05 (0-0.17)	0 (0-0.03)	0.033
Species	*AM420091_s* ↑	0.24 (0-1.94)	1.53 (0.17-10.64)	0.043
	Streptococcus salivarius ↓	3.22 (0.76-10.42)	0.71 (0.03-3.84)	0.007
	Streptococcus parasanguinis↓	0.98 (0.24-8.42)	0.33 (0.03- 1.11)	0.043
	Capnocytophaga gingivalis ↑	0.19 (0.04- 0.62)	0.73 (0.04- 3.5)	0.009
	Neisseria flava ↑	0 (0-0.1)	0.32 (0-6.99)	0.003
	Rothia dentocariosa ↓	0.97 (0.06-2.31)	0.28 (0-0.69)	0.043
	HQ767899_s ↓	0.44 (0.02-2.97)	0.04 (0-1.29)	0.034
	Aggregatibacter segnis ↑	0.03 (0-0.19)	0.28 (0-2.65)	0.023
	Capnocytophaga sputigena ↑	0.05 (0-0.2)	0.36 (0.06-1.62)	0.003
	4P003196_s ↑	0.04 (0.01-0.42)	0.14 (0.1- 2.09)	0.021
	Neisseria sicca group ↓	0.25 (0-6.62)	0 (0-0)	< 0.0005
	Actinomyces viscosus ↓	0.05 (0-1.37)	0 (0-0.16)	0.028
	HQ757980_s ↓	0.1 (0.03-0.88)	0 (0-0.74)	0.032
	4P004176_s ↑	0 (0-0.03)	0.04 (0-0.23)	0.015
	Porphyromonas catoniae ↑	0 (0-0.01)	0.03 (0-0.23)	0.029
	Escherichia coli group ↓	0.06 (0-0.17)	0 (0-0.03)	0.033
	AM420214_s ↑	0 (0-0.01)	0.01 (0- 0.13)	0.021
